# NUMB suppression by miR-9-5P enhances CD44^+^ prostate cancer stem cell growth and metastasis

**DOI:** 10.1038/s41598-021-90700-x

**Published:** 2021-05-27

**Authors:** Xuan Wang, Jun Cai, Lei Zhao, Dejun Zhang, Guojie Xu, Jianli Hu, Tao Zhang, Min Jin

**Affiliations:** 1 Cancer Center, Union Hospital, Tongji Medical College, Huazhong University of Science and Technology, Wuhan, 430022 China; 2 Wuhan Pulmonary Hospital, Wuhan Institute for Tuberculosis Control, Wuhan, 430030 Hubei China; 3 Department of Oncology, First Affiliated Hospital of Yangtze University, Jingzhou, 434000 Hubei China

**Keywords:** Cell migration, Cancer stem cells, Metastasis

## Abstract

Experimental and clinical studies over the past two decades have provided overwhelming evidence that human cancers, including prostate cancer (PCa), harbor cancer stem cells (CSCs) that sustain tumor growth, drive tumor progression and mediate therapy resistance and tumor relapse. Recent studies have also implicated NUMB as a PCa suppressor and an inhibitor of PCa stem cells (PCSCs); however, exactly how NUMB functions in these contexts remains unclear. Here, by employing bioinformatics analysis and luciferase assays and by conducting rescue experiments, we first show that NUMB is directly targeted by microRNA-9-5p (miR-9-5p), an oncogenic miR associated with poor prognosis in many malignancies. We further show that miR-9-5p levels are inversely correlated with NUMB expression in CD44^+^ PCSCs. miR-9-5p reduced NUMB expression and inhibited numerous PCSC properties including proliferation, migration, invasion as well as self-renewal. Strikingly, overexpression of NUMB in CD44^+^ PCSCs overcame all of the above PCSC properties enforced by miR-9-5p. Taken together, our results suggest that inhibiting the expression of the oncomiR miR-9-5p and overexpressing NUMB may represent novel therapeutic strategies to target PCSCs and PCa metastasis.

## Introduction

Prostate cancer (PCa) is one of the most commonly diagnosed male cancers worldwide^1^, with an estimated 1.6 million cases and > 360,000 deaths each year^2^. PCa patients are treated with surgery (i.e., radical prostatectomy) or endocrine, chemo- or radio-therapies. Many PCa patients treated with endocrine blockade initially demonstrate androgen-dependent (AD) disease, which eventually becomes androgen-independent (AI) or castration-resistant, for which there is no effective treatment, and patients frequently succumb to metastasis and death^3^. Several new drugs have recently been developed for the treatment of Castration-Resistant Prostate Cancer (CRPC), including enzalutamide, androgen synthesis inhibitors (Abiraterone and TAK-700), androgen receptor antagonists (MDV3100) as well as immunotherapy^4–8^; nevertheless, patient tumors rapidly develop resistance and overall survival is only marginally improved by a few months. Therefore, it is of paramount importance to elucidate the cellular and molecular mechanisms underlying therapy resistance and CRPC development.

Overwhelming evidence in the past two decades has demonstrated that human cancers harbor cancer stem cells (CSCs) that are likely responsible for developing resistance to conventional treatment and tumor reccurence^9,10^. CSCs in treatment-naïve tumors generally constitute a relatively minor fraction; however, these CSCs possess many hardcore stem cell properties and may perpetuate infinite tumor growth. One of the best-studied CSC populations is the CD44^+/hi^ population, which has been widely reported in solid tumors including cancers of the prostate, breast, pancreas, head and neck, and ovary^11,12^. Consequently, an in-depth understanding of prostate CSCs (PCSCs) may undercover novel molecular regulators and provide novel anti-tumor strategies.

NUMB was originally identified in *Drosophila* and is an evolutionarily conserved protein widely expressed in mammalian tissues that has a wide variety of functions such as cell fate determination, asymmetric self-renewal, cell division, proliferation, migration and other signaling pathways^13^. Recent studies suggest that NUMB may have a tumor-suppressive role in various tumor types^14–16^. Interestingly, NUMB expression is highly heterogeneous in tumors and may be specifically reduced in CSCs^15–17^. In PCa, NUMB^-/low^ PCa cells behave as PCSCs that resist androgen-deprivation therapy (ADT) and preferentially express stem cell-related genes^17–19^. However, how NUMB is downregulated in PCa and becomes depleted in PCSCs remains unclear.

microRNAs (miRNAs) can regulate gene expression via binding to the 3'-untranslated region (UTR) of target mRNAs, and miRNAs have been reported to regulate the biological properties of various CSCs^20^. For example, our group previously demonstrated that miR-128 could inhibit PCSC characteristics via directly targeting the stemness factor BMI-1^21^. Likewise, miR-34a may directly inhibit early-stage colon CSC (CCSC) self-renewal and differentiation by directly repressing NUMB expression^22^. In PCa and PCSCs, it remains unknown whether miRNAs may mediate NUMB downregulation. Of interest, miR-9 was recently found to be differentially expressed in multiple cancer cells as well as in CSCs, and miR-9 appears to causally regulate cancer development^21–24^. In particular, miR-9-5p is a newly identified onco-miR that promotes PCa progression^25,26^. Here, we test the hypothesis that miR-9-5p may enforce PCSC properties and promote PCa aggressiveness via directly targeting NUMB. Our results indicate that miR-9-5p reduced NUMB expression and inhibited numerous PCSC properties including proliferation, migration, invasion as well as self-renewal. Strikingly, overexpression of NUMB in CD44^+^ PCSCs overcame all of the above PCSC properties elicited by miR-9-5p.

## Results

### NUMB has an inhibitory role in CD44+ PCSCs

**Figure 1:**
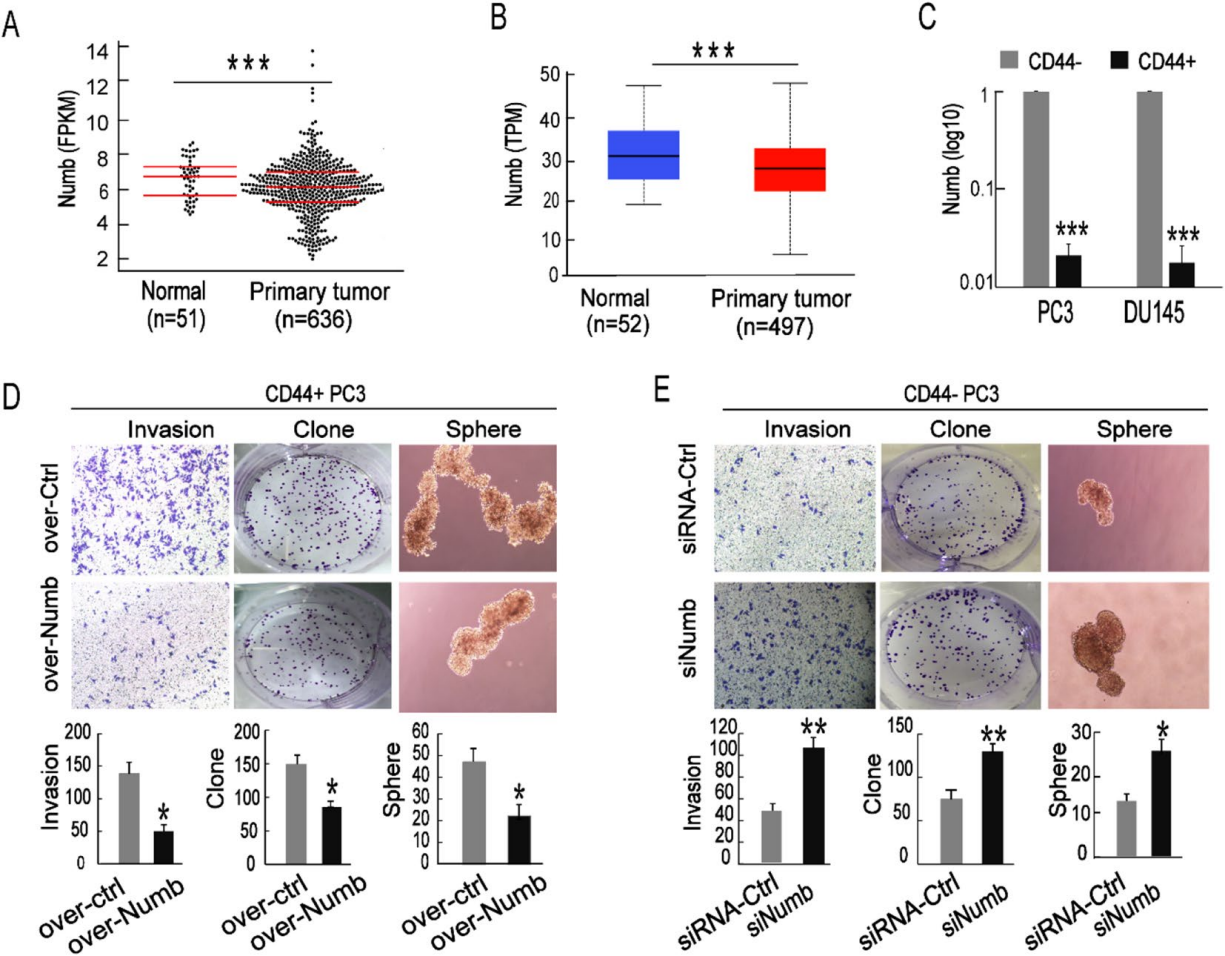
NUMB has an inhibitory role in PCa and PCSCs. (**A**,**B**) The TCGA database and StarBase v3.0 (http://starbase.sysu.edu.cn/) were analyzed to detect expression differences of NUMB genes in normal tissues and primary tumors (FPKM = Fragments Per Kilobase Million, TPM = Transcripts Per Million). (**C**) qRT-PCR was used to detect NUMB expression differences in CD44+ and CD44− PC3 cells. (**D**) Transwell invasion, clone formation and sphere formation assays were performed with CD44+ PC3 cells transfected with pcDNA3.1-Numb (Over-Numb) or pcDNA3.1-Ctrl (Ctrl-Numb). (**E**) Transwell invasion, colony formation and sphere formation assays were performed with CD44- PC3 cells transfected with siRNA-Numb (SiNumb) or the control (siRNA-Ctrl).

We separately used the TCGA platform and StarBase 3.0 database to compare the expression of NUMB in normal prostate tissue and PCa tissue. We found that the expression was significantly downregulated in PCa tissues compared with normal tissues (Fig. 1A,B). To detect the expression in PCSCs, we isolated CD44-positive (CD44+) PCa cells representing PCSCs and CD44-negative (CD44−) PCa cells representing non-PCSCs. Through qRT-PCR, we found that compared with the CD44− PCa cells, NUMB expression was significantly decreased in both CD44+ PC3 and DU145 cells (Fig. 1C). To address the role of NUMB in CD44+ PCSCs, we altered the expression of NUMB in the CD44+ and CD44− PC3 cells and then performed tumor invasion, clone formation and sphere formation assays. As expected, when CD44+ PC3 cells were transfected with the NUMB overexpression plasmid (pcDNA3.1-NUMB), the mean number of cells that invaded through the Transwell membrane was significantly decreased (160 ± 18 vs. 50 ± 9), clone formation was markedly attenuated (150 ± 14 vs. 82 ± 12), and spheroid formation in serum-free medium was obviously suppressed (48 ± 15 vs. 16 ± 6); moreover, cell morphology was much smaller compared to the respective controls. (Fig. 1D). In contrast, when CD44− PC3 cells were transfected with siRNAs targeting NUMB, the number of cells that invaded through the Transwell membrane was significantly increased, cell clone formation was significantly enhanced, and the number and size of spheroids formed in serum-free medium was increased compared to the respective controls (Fig. 1E). These results showed that NUMB exerted anti-cancerogenic effects on PCa and PCSCs and that the stemness characteristics of PCSCs were at least in part promoted by NUMB.

### MiR-9-5p showed an opposite expression pattern compared to NUMB in PCa and directly bound NUMB

**Figure 2:**
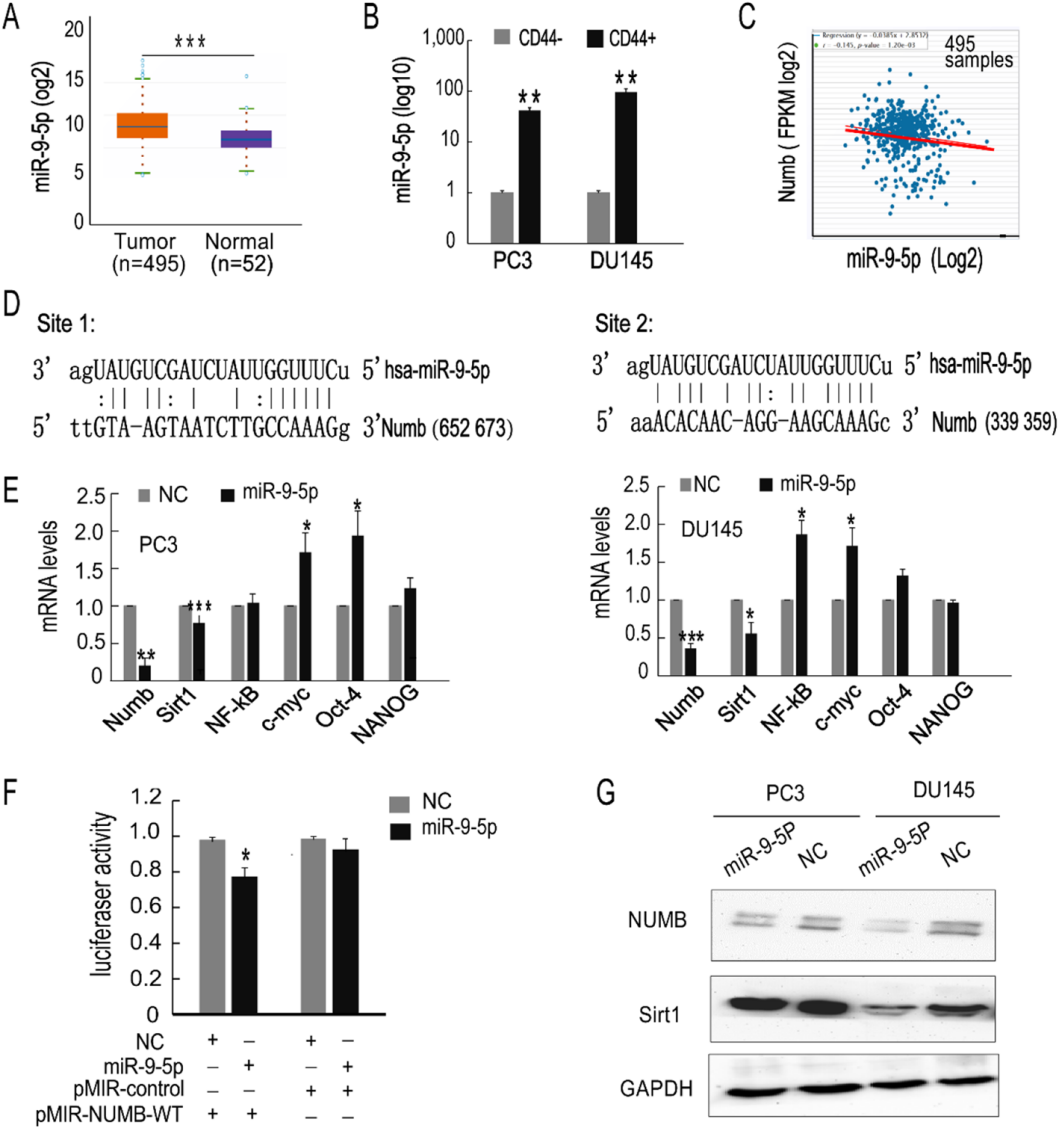
MiR-9-5p regulates the expression of Numb and several stemness-related genes. (**A**) StarBase 3.0 was used to detect differences in the expression in normal tissue and primary tumors. (**B**) Detection of the difference in miR-9-5p expression in CD44+ and CD44− PC3 cells. (**C**) The TCGA database was used to detect the relationship between Numb and miR-9-5p in PCa patients (FPKM = Fragments Per Kilobase Million). (**D**) Prediction of miR-9 binding sites in Numb using miRanda. (**E**) Detection of the expression of Numb/Notch 1 signaling pathway-associated and stemness molecules after overexpression of miR-9-5p in PC3 and DU145 cells. (**F**) A luciferase reporter assay was performed to confirm the direct binding of miR-9-5p in the NUMB 3′-UTR in PC3 cells. (G) Western blotting was used to detect Numb and Sirt1 expression in PC3 and DU145 cells overexpressing miR-9-5p and the experiments were repeated 3 times by using independent sample sets. *P < 0.05, **P < 0.01, ***P < 0.001.

To explore the upstream regulatory mechanism, we examined the miRNA-mRNA network. Among various miRNAs, miR-9-5p, which has been demonstrated to be an onco-miRNA in PCa, was selected. Using StarBase 3.0, we found that miR-9-5p expression was upregulated in PCa tissues compared with normal tissues (Fig. 2A), and miR-9-5p showed an opposite expression pattern compared to NUMB in PCa (Fig. 2C). Subsequently, we explored the expression levels in CD44+ PCSCs. As shown in Fig. 2B, CD44+ PCSCs displayed higher miR-9-5p expression levels than CD44− PCa cells. Using miRanda, we identified two possible NUMB-miR-9-5p binding sites. (Fig. 2D). In addition, overexpression of miR-9-5p in the PC3 and DU145 cells changed the mRNA levels of different stem-related molecules. The mRNA levels of NUMB and another tumor suppressor, Sirt1, were downregulated in both cell lines following miR-9-5p overexpression; furthermore, c-myc and Oct-4 were significantly upregulated in PC3 cells, while NF-κb and c-myc were significantly upregulated in DU145 cells (Fig. 2E). Moreover, a luciferase reporter assay was carried out to test the interaction between NUMB and miR-9-5P. The data indicated that miR-9-5P mimic transfection significantly attenuated the luciferase activity of the vectors containing the wild-type NUMB 3'-UTR, while no obvious luciferase activity effect was observed in the control mimic-transfected group (Fig. 2F). Western blot results also consistently showed that the NUMB protein was decreased in PCa cells overexpressing miR-9-5p. In addition, Sirt1 protein levels were reduced when PCa cells were transfected with miR-9-5p (Fig. 2G). The detailed blots and three repeated assays by using independent sample sets for NUMB, Sirt1 and the internal reference of GAPDH expression are presented in Supplementary Figure S1. The gene expression results were validated by an mRNA array in miR-9-5p-upregulated PC3 cells (Supplementary Figure S2). Taken together, these results demonstrated that NUMB and miR-9-5p showed opposite expression patterns in PCSCs, and overexpression of miR-9-5P suppressed NUMB expression via binding to its 3'-UTR.

### MiR-9-5p has a cancerogenic role in PCa cells

**Figure 3:**
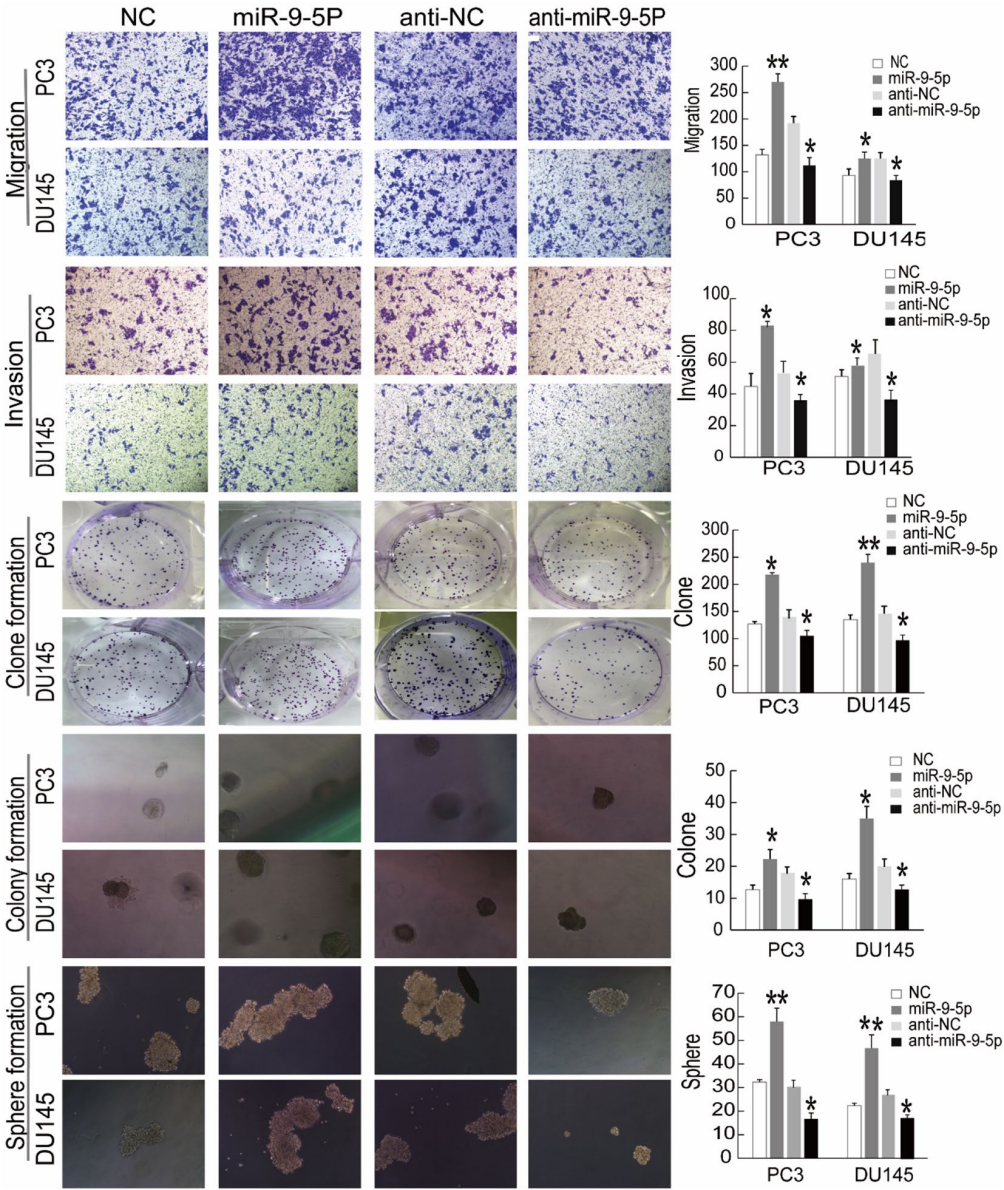
MiR-9-5p was significantly associated with PCa cell invasion, migration, and clonal, clonogenic and sphere-forming activities in vitro. From the upper part to the lower part; Transwell migration assays, invasion assays, clonal experiments, 3-D Matrigel clonogenic assays and sphere formation assays were separately performed to determine the metastasis and stemness characteristics of PCa cells. PC3 and DU145 cells were transfected with miR-9-5p mimic, mimic NC, miR-9-5p inhibitor or inhibitor NC for 48 h. All results are shown as the mean ± S.D. from three independent experiments. Statistical analysis was carried out using GraphPad Prism 7.0 Software (GraphPad Software, La Jolla, CA). *P < 0.05, **P < 0.01.

To assess the biological function of miR-9-5p in PCa, we performed in vitro gain- and loss-of-function analyses of miR-9-5p in bulk DU145 and PC3 cells. Exogenously introduced miR-9-5p significantly increased PCa cell migration, invasion, clone establishment, 3-D Matrigel colony establishment and sphere formation number (Fig. 3, left). Contrary to previous results, exogenously inhibiting miR-9-5p expression resulted in the opposite findings. In the miR-9-5p inhibitor-transfected group, PCa cell migration, invasion, clone establishment, 3-D Matrigel colony establishment and sphere formation were decreased compared in the NC groups (Fig. 3, right). Taken together, these series of assays indicated that miR-9-5p exerted a cancerogenic role on clonal and clonogenic abilities and CSC-associated stemness properties.

### MiR-9-5P promotes the stemness characteristics of CD44+ PCSCs

**Figure 4:**
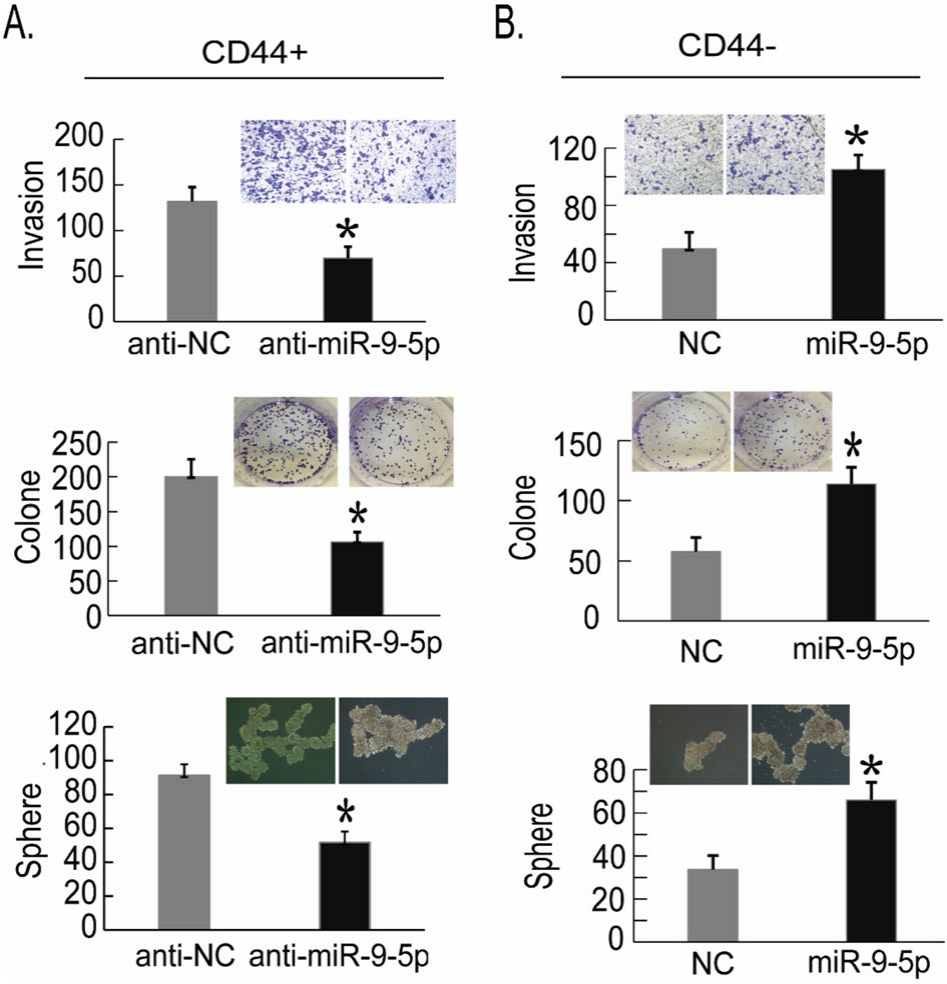
MiR-9-5P has a promoting role in CD44+ PCSCs. (**A**) After sorting of CD44+ and CD44− PC3 PCa cells by flow cytometry, decreased expression of miR-9 in the CD44+ PC3 cells was mediated by the transfected miR-9 inhibitor, and the invasion, clone formation and sphere formation abilities of CD44+ PC3 cells were detected 48 h later. (**B**) Sorting of CD44+ and CD44− PC3 PCa cells was performed by flow cytometry, and high expression of miR-9-5p was detected in the CD44− PC3 cells transfected with the miR-9-5p mimic. After 48 h, the invasion, clone formation and sphere formation abilities of CD44− PC3 cells were detected. The results are represented as the mean ± S.D. from three independent assays. *P < 0.05.

As the expression levels were substantially increased in CD44+ PCa cells (Fig. 2B), we hypothesized that miR-9-5p has a pro-oncogenic role in PCSCs, in contrast to NUMB. To illustrate this, we manipulated the expression levels of miR-9-5p in CD44+ PCSC and CD44− non-PCSC PC3 cells by transfection with miR-9-5p mimic or inhibitor followed by cell invasion, clonal and sphere-forming assays (Fig. 4). These assay results demonstrated that transfection of miR-9-5p inhibitors in purified CD44+ PC3 cells obviously restrained PCSC cell invasion, clonal formation and sphere formation because there were fewer regenerations and developing spheres were smaller than those in the control group (Fig. 4A). Groups transfected with the miR-9-5p mimic in the purified CD44− PC3 cells exhibited more invasive cells, more clonal formation and more sphere formation than the control groups (Fig. 4B). Collectively, these results indicated that miR-9-5p promoted the pro-oncogenic characteristics of CD44+ PCSCs.

### NUMB is a functional target of miR-9-5p in CD44+ PCSCs

**Figure 5:**
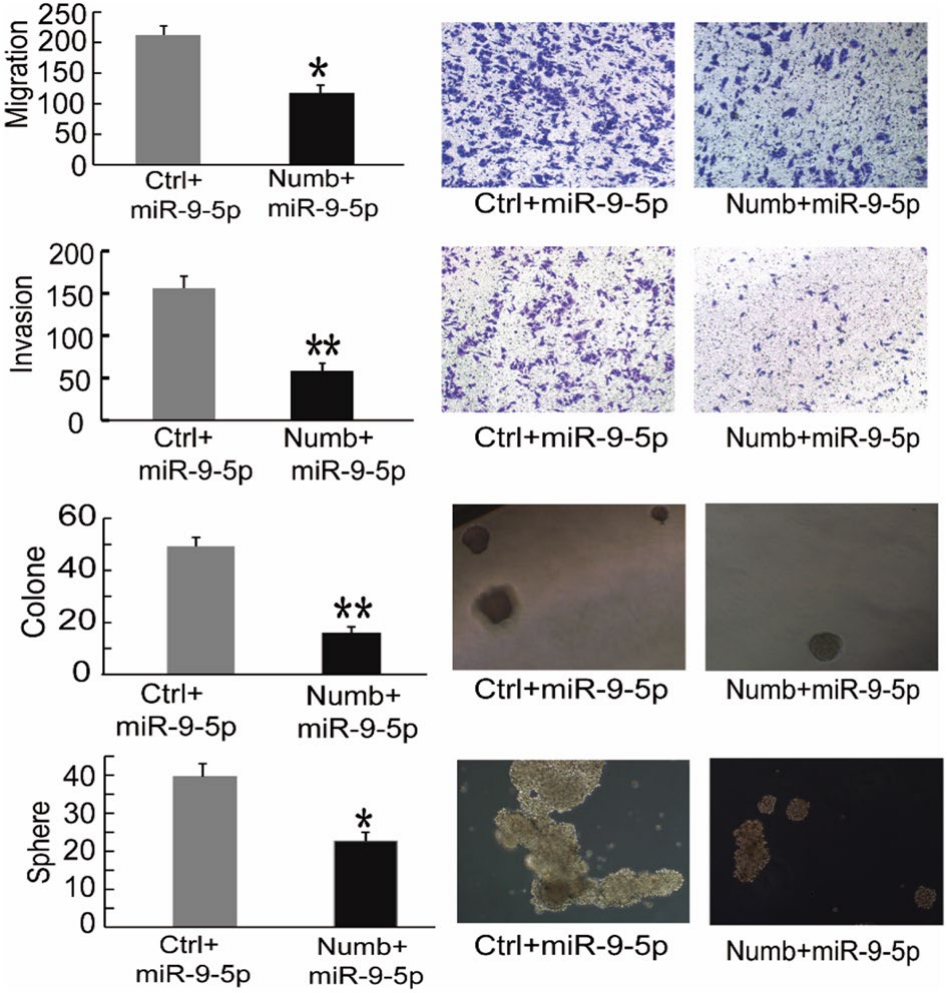
NUMB is a functional target of miR-9-5p in PCa cells. PC3 cells cotransfected with miR-9-5p mimic and NUMB cDNA lacking the NUMB 3'-UTR restored the expression of NUMB after 48 h of culture. From the upper part of the figure to the lower part of the figure, the migration, invasion, clone formation and sphere formation abilities of the cotransfected PC3 cells were detected. The results are represented as the mean ± S.D. from three independent assays. *P < 0.05, **P < 0.01.

To verify whether NUMB is a functional target of miR-9-5p, we performed rescue assays. A plasmid expressing the NUMB gene containing all cDNA sequences but excluding the 3'-UTR sequences (NUMB-ORF) was constructed. Therefore, this NUMB overexpression plasmid could not bind to miR-9-5p. We then cotransfected plasmids containing the NUMB-ORF sequences and the miR-9-5p mimic into CD44+ PC3 cells. The results showed that PCSCs cotransfected with NUMB and miR-9-5p mimic showed decreased migration, invasion, 3-D Matrigel colony establishment and sphere formation compared with cells cotransfected with empty vector and miR-9-5p mimic (Fig. 5). In conclusion, these results indicated that NUMB is a functional target that alters the invasive and tumorigenic effects of miR-9-5p in PCSCs.

### MiR-9-5p promotes xenograft tumor growth and metastasis in vivo

**Figure 6:**
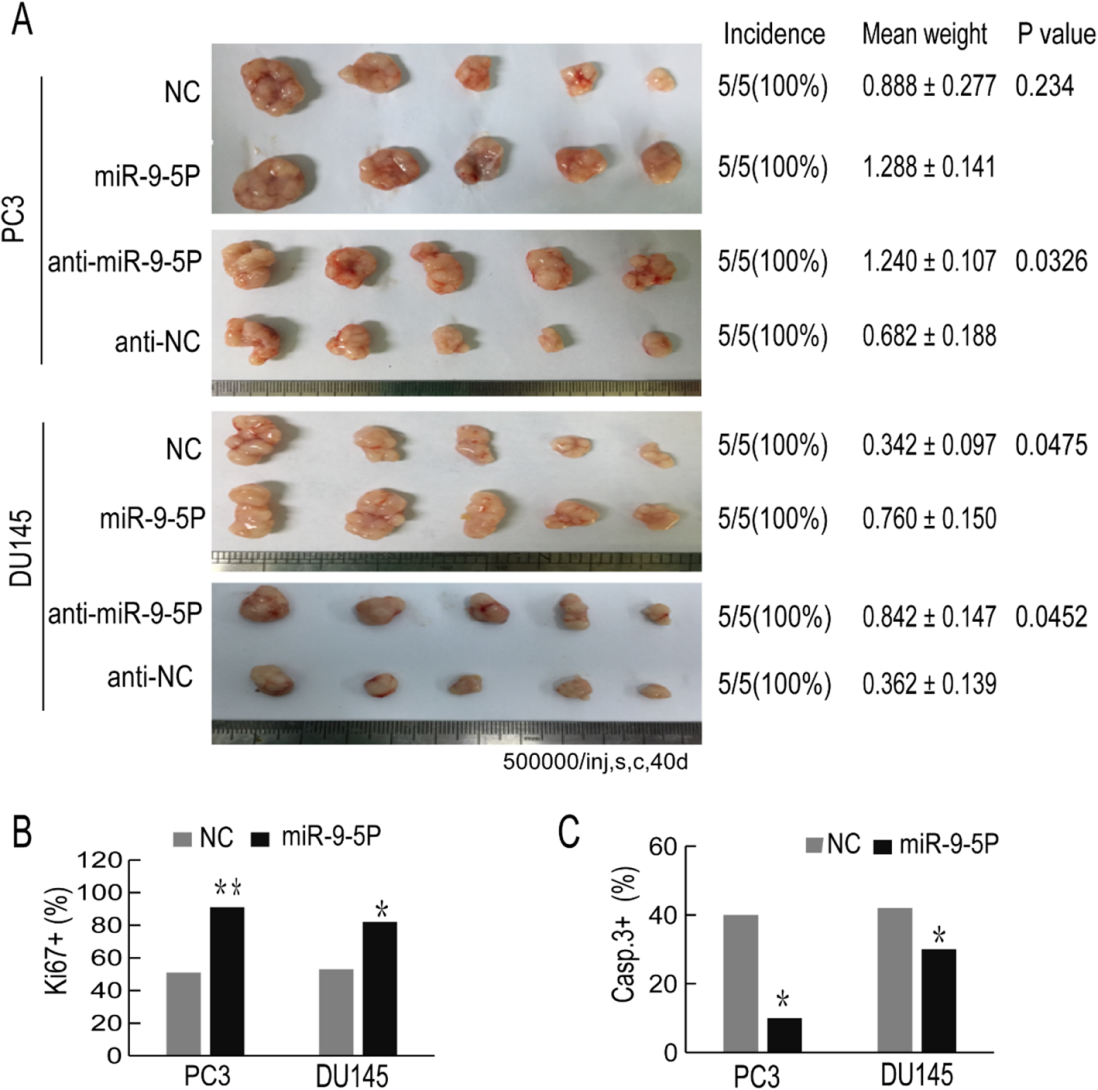
Overexpression of miR-9-5p promotes xenograft tumor growth in vivo. (A) Overexpression or decreased expression of miR-9-5p in PC3 or DU145 cells was generated, after which the transfected cells were subcutaneously injected into BALB/C nude mice. Tumors were harvested and the tumor images is shown on the left of the picture. The incidence (tumors/injections) and weight of the tumors (mean ± S.D.) and the corresponding *P* values are shown on the right of the picture. (B, C) Representative immunohistochemical (IHC) staining pictures are shown, in which the Ki-67- or Caspase-3-positive cells from the harvested tumor tissues were stained (magnification, × 200). The percentage of Ki67-positive or Caspase-3-positive cells was calculated. All results are represented as the mean ± SEM from three independent assays. *P < 0.05, **P < 0.01.

To further investigate the effects of miR-9-5p on tumorigenesis, we transfected chemically synthesized miR-9-5p mimic or inhibitor oligos (30 nM, 48 h) into PCa cells (PC3 and DU145 cells). Forty-eight hours after transfection, BALB/C nude mice (8-week-old) were s.c. implanted with transfected PCa cells. Notably, PC3 cells that were transferred to the mice could significantly promote tumor growth. Although the DU145 transfection group showed no significant differences in tumor growth, the mean tumor weight was substantially increased (Fig. 6A). In contrast, downregulation of miR-9-5p in both the PC3 and DU145 cells inhibited tumor growth. We also conducted lung metastasis assays via a tail vein injection model in vivo using miR-9-5P-overexpressing PC3 cells. The data (Supplementary Figure S3) showed that miR-9-5p facilitated tumor metastasis compared with the control. Then, some harvested subcutaneous tumors were used for immunohistochemical staining to detect Ki67 and Caspase-3 expression levels in the s.c. xenograft tumors. The percentage of Ki67 + cells increased in the miR-9-5p mimic-transfected groups (Fig. 6B), while the percentage of Caspase-3 + cells was reduced in the miR-9-5p mimic-transfected groups (Fig. 6C). These results confirmed that the tumorigenic effects of miR-9-5p may accelerate cell proliferation and restrain cell apoptosis. Combined with the above results, miR-9 acts as a carcinogenic regulator both in vivo and in vitro.

## Discussion

There is an urgent need to identify novel, mechanism-driven treatments for advanced PCa, and, in particular, CRPC^3^. CSCs, generally representing a small proportion of the bulk in treatment-naive tumors, are known to be refractory to radiation and chemotherapeutic drugs and represent the main source of tumor recurrence and metastasis^9^. Many CSCs have been enriched and isolated using the cell surface protein CD44^11^, either alone or combined with other markers such CD133^27^, integrin^28^ and ALDH1A^29^. CD44^+^ PCSCs have been considered as "evil seeds" that are naturally resistant to hormone therapy and endow enhanced clonogenic, tumorigenic and metastatic capabilities^26^. Therapeutic targeting and elimination of PCSCs remain a challenging goal in our efforts to prevent CRPC and improve patient survival.

Recent studies have revealed prevalent deletion of *NUMB* in various cancers^14–16^, and loss of NUMB has been implicated in promoting tumor growth, invasion, metastasis, and stemness^14,16–19,27^. NUMB acts as a tumor suppressor via modulating several important signaling pathways including P53, NOTCH and Hedgehog^14,28^. As a critical cell-fate determinant during development, NUMB also appears to regulate division mode and fate in CSCs. In PCa, NUMB is negatively associated with tumor progression^16^, and reduced NUMB expression has been observed in castration-resistant PCSCs. In our current study, we have also observed diminished NUMB expression in PCa compared to normal tissues, and substantially downregulated NUMB in CD44^+^ PCSCs (compared to CD44− non-PCSCs). Critically, overexpression of NUMB in CD44^+^ PCSCs inhibited their invasiveness and clonal and sphere formation capabilities. Collectively, our data support that NUMB expression is reduced in PCa and NUMB negatively regulates the biological properties of PCSCs.

Many factors, such as promoter hypermethylation and gene mutation, may result in low NUMB expression in PCa, although the exact mechanism remains unknown. miRNAs in the human genome regulate hundreds of thousands of genes to control a multitude of biological processes and, importantly, some miRNAs have been reported to regulate CSC stemness^20^. miR-9 was first identified as a critical modulator in neurogenesis and nerve tissue development^33^, and interestingly, the expression and function of miR-9 appear to be dysregulated in many cancers, but in a cancer type dependent manner. For instance, miR-9 acts as an oncomiR in breast and non-small cell lung carcinomas, whereas it appears to exert tumor-suppressive functions in ovarian cancer and glioblastoma multiforme^30–33^. In PCa, Seashols-Williams SJ showed that miR-9 promotes PCa cell growth by targeting E-cadherin and SOCS5^34^, and a subsequent study revealed that miR-9 promotes epithelial mesenchymal transformation (EMT) of PCa cells by regulating StarD13^35^. We have observed that miR-9-5p levels are upregulated in PCa, especially in CD44^+^ PCSCs, and that miR-9-5p expression promoted proliferation, clonal formation, migration, and invasion of bulk PCa cells. Furthermore, miR-9-5p enhanced the tumorigenic and metastatic capabilities of PCSCs, which is in stark contrast to NUMB’s functions in PCSCs. Several pieces of evidence then indicated that miR-9-5p exerted its PCa-promoting functions, at least partly, by targeting NUMB. *FIRST*, NUMB is downregulated in the mRNA array of miRNA-9-5p-overexpressing PCa cells. *SECOND*, bioinformatics analyses revealed NUMB as a target gene of miR-9-5p via two binding sites at the NUMB 3'-UTR. *THIRD*, luciferase reporter assays validated the relevance of the two binding sites. *FINALLY*, the effects of miR-9-5p on PCSC proliferation, metastasis and self-renewal were partially rescued by restoring NUMB expression. Altogether, these data demonstrate that miR-9-5p functions as an oncomiR through NUMB suppression and may represent a therapeutic target in PCa.

## Conclusion

Our present study has defined NUMB as a novel direct target of oncomiR miR-9-5P in CD44^+^ PCSCs. miR-9-5P inhibition or NUMB overexpression can attenuate many biological properties of PCSCs, leading to prominent inhibition of clonogenic and sphere-forming activities, tumor growth and metastasis. Our results, in aggregate, suggest that the newly identified miR-9-5p/NUMB pathway may be a potential therapeutic target for treatment of PCa and PCSCs.

## Materials and methods

### Cells, animals, and reagents

PC3 cells were cultured in DMEM, and DU145 cells were cultured in 1640 medium (Gibco). Ten percent fetal bovine serum (FBS) and 1% antibiotic were added to both the DMEM and 1640 media. BALB/c mice were purchased from the Beijing Weitong Lihua Company, and the ethics committee approved all animal experiments. Prestained Protein Ladder (Thermo Fisher Scientific, #26,617) and antibodies against Ki-67 (polyclonal, Servicebio, #GB111141), Caspase-3 (polyclonal, Servicebio, No. GB1100-1), GAPDH (polyclonal, Abclonal, #AC002), NUMB (polyclonal, Abcam, #Ab14140) and Sirt1 (polyclonal, Abclonal, #A0230) were used.

### Oligonucleotides (oligos), plasmids, and transfection

miRNA mimics (Sequence: UGAAAGCCAAUAGAUCGAAAUA) enhance the function of endogenous miRNAs by chemical synthesis. miRNA inhibitors (Sequence: UCAUACAGCUAGAUAACCAAAGA) are chemically modified inhibitors targeting one specific miRNA. miR-9-5p mimics and inhibitors were obtained from Gemma Company in Shanghai. Lipofectamine RNAiMAX reagent (Invitrogen, #13,778–150) was utilized to transfect PC3 and DU145 cells with 30 nmol miR-9-5p mimic or inhibitor, as well as the NC. NUMB siRNA for knockdown of NUMB expression and its control (NC) were transfected into cells with RNAiMAX. The NUMB overexpression plasmid (pcDNA3.1-NUMB) containing the full-length gene was transfected with Lipofectamine 2000 (Invitrogen, #11,668–019). The NUMB gene cDNA is based on the pcDNA 3.1-Ctrl empty vector. Cotransfection of the PmirGLO-NUMB plasmid lacking the NUMB 3'-UTR and its control PmirGLO (Ctrl) with the miR-9-5p mimic was performed for rescue experiments. miR-9-5p mimic or NC transfection with the pMIR-control vector and the pMIR- miR-9-WT plasmid was used for the luciferase reporter assays.

### Bioinformatics analyses and mRNA array

The TCGA online platform and StarBase software were employed to explore NUMB expression in PCa tissues and matched normal tissues. The expression in PCa and the relationship between NUMB and miR-9-5p expression in PCa were also analyzed using StarBase. For mRNA-seq, microarray analysis was performed by Genminix Informatics Co., Ltd. (Shanghai, China), in which six cell samples (three cell samples transfected with miR-9-5p mimic and three control cell samples transfected with control mimic) were used for the mRNA microarray analysis.

### Transwell migration, invasion and lung metastasis model

A mixture of matrix gel (BD, #354,263) and serum-free medium was added to each well on ice. The sample was incubated overnight. The above steps were performed for the Transwell invasion assay but not the Transwell migration assay. Cells were transfected two days before the experiment and counted 48 h after digestion. The cell concentration was adjusted to 1 × 10^6^ cells per ml. Six hundred microliters of medium with 10% FBS was incubated in the lower cavity, and 200 µl of serum-free medium was incubated in the upper cavity. After 36–48 h in an incubator, the cells were fixed with polyformaldehyde for 30 min and stained with crystal violet. The cells were carefully wiped away with a cotton swab. When the chamber was dry, photos were taken under an inverted microscope, and the cells were finally counted. For establishment of the lung metastasis model, the cell concentration was adjusted to 4 × 10^7^ cells per ml. BALB/C nude mice (5-week-old) were injected with 100 µl of cell suspension (4 × 10^6^ cells) through the tail vein. The changes in the mice were observed every 3–4 days. Moreover, 40-day-old mice were sacrificed, their lungs were isolated, and the number of metastatic nodules was observed and counted.

### Clonal, clonogenic, and sphere-formation assays

These detailed methods are described in our previous article^21^. For the clonal experiments, PCa cells were inoculated into a 6-well plate at an appropriate density (500 or 300 cells per well). When obvious clones appeared, they were counted within 14 days. For the clonogenic assays, the cell concentration was adjusted to 5 × 10^3^ cells per ml. Sixty microliters of Matrigel and 60 μl of cell suspension containing 300 cells were mixed at a ratio of 1:1 and then added carefully around the edge of a 24-well plate. After, solidification for 20 min at 37 °C, 400 μl of medium with serum was added. The cells were counted 7–14 days after culture. For the sphere formation assay, 300–500 cells per well were inoculated in ultralow attachment 6-well plates containing F12/DMEM medium supplemented with fibroblast growth factor (FGF, #PHG0026), epidermal growth factor (EGF, #PHG0313) and 1 × B27. The cells were counted and photographed within 14 days.

### Flow cytometry analysis

PCa cells (500,000) were aliquoted into assay tubes, 2–3 ml of incubation buffer was added, and the tubes were rinsed by centrifugation. The cells were resuspended in 90 µl of incubation buffer and blocked at room temperature (RT) for ten minutes. Ten microliters of CD44-PE conjugated antibody (#FAB36609) was added to each tube and incubated for 30–60 min in the dark at RT. The cells were washed followed by centrifugation. The cells were resuspended in 500 µl of PBS and analyzed by FACS. The CD44 positive and negative population cells were collected by sorting.

### Tumor transplantation experiments and immunohistochemistry

For subcutaneous tumorigenesis experiments, we transfected PC3 and DU145 cells with miR-9-5p mimic or inhibitor and the negative control. After quantification, the cell concentration was adjusted to 5 × 10^6^ per ml. BALB/C nude mice were subcutaneously (s.c.) injected with 100 μl of the cells. When the subcutaneous tumor volumes approached 80–100 mm^3^, the mice were killed. After subcutaneous tumorigenesis, the tumors were embedded, sectioned and stained. Caspase-3 was detected in the cytoplasm and Ki67 was detected in the nucleus; positive staining appeared brown and yellow, respectively. Typical lung metastases were selected for HE staining and photography.

### Immunohistochemical analysis

Immunohistochemical analysis was carried out after immobilization in polyformaldehyde. The subcutaneous tumors were embedded, sectioned and stained. Caspase-3 and Ki67 were detected in the cytoplasm and nucleus, respectively. Brown and yellow colors indicate positive expression, respectively. Representative lung metastases were chosen for HE staining and photography.

### Western blot

Cell lysis was performed with lysis buffer. The total protein of the cells was extracted, and the concentration of the protein was measured. We carried out polyacrylamide gel electrophoresis to separate the proteins according to their molecular size, then the proteins were transferred from the gels to polyvinylidene fluoride (PVDF) membranes. Next, we cut the corresponding PVDF bands at the proper molecular weight size for the three target protein (NUMB, Sirt 1 and GAPDH) via the prestained protein marker guilding. Subsequently, we made a triangular cut on the left upper corner of each band to mark the sample order. Following this, the membranes bands were blocked and incubated at 4 °C overnight with corresponding primary antibodies. After washing three times, the membranes bands were incubated for 2 h using corresponding secondary antibodies at RT. Then, ECL reagent was used for imaging the blots. GAPDH was utilized as an internal reference. The protein results are expressed as relative quantification. Primary rabbit antibodies against NUMB (1:1000 dilution, polyclonal) was purchased from Abcam. The primary rabbit antibodies against Sirt1 (1:1000, polyclonal), and GAPDH (1:5000 dilution, polyclonal) were provided by Abclonal. The secondary anti-rabbit HRP-linked antibody (1:3000 dilution) was purchased from Servicebio. The Western Blot analysis were repeated 3 times separately by using independent sample sets.

### Real-time PCR

Total RNA was extracted from the tested cells with the TRIzol reagent (Invitrogen) according to the protocol. Quantitative real-time PCR (qRT-PCR) was conducted according to the kit instructions. The qRT-PCR primers were synthesized by Wuhan Genecreate Company (China). RNA was used to generate cDNA, and then the cDNA was amplified according to a standard protocol. GAPDH or U6 was used as an internal reference. The results are shown as 2^−ΔΔCT^. Each experiment was independently repeated three times. The primers used in this study are listed in Table 1.

**Table 1:** Primer sequences.

Primer name	Sequence
Numb forward primer	AACGCCAACTATCCCTAGG
Numb reverse primer	ACTGGTTTGGTCATCGGAG
Sirt1 forward primer	GCTGGCCTAATAGAGTGGCAA
Sirt1 reverse primer	CTCAGCGCCATGGAAAATG
NF-kB forward primer	GCACCCTGACCTTGCCTATT
NF-kB reverse primer	AGCTGCTTGGCGGATTAG
c-Myc forward primer	AGGCTCCTGGCAAAAGGT
c-Myc reverse primer	CTGCGTAGTTGTGCTGATGTG
Oct-4 Forward primer	GCCAGAGGAAAGCACACT
Oct-4 reverse primer	CAGATCAGCCACARCGC
NANOG forward primer	TCTTCTGCTGAGATGCCTCAC
NANOG reverse primer	GGTTGTTTGCCTTTGGGAC
GAPDH forward primer	AGAAGGCTGGGGCTCATTTG
GAPDH reverse primer	AGGGGCCATCCACAGTCTTC

### Statistical analysis

All experiments were independently repeated three times, and the results are expressed as the mean ± S.D. The Student’s t-test was used to compare the differences between the experimental group and the control group. The data were considered significantly different at a *P* value < 0.05. The data and graphs were generated using the GraphPad Prism 7.00 software.

### Study approval

The study design was approved by the Ethics Committee of Union Hospital affiliated to Tongji Medical College of Huazhong University of Science and Technology, and all experiments were performed in accordance with relevant guidelines and regulations. This study was carried out in compliance with the ARRIVE guidelines.

## Supplementary Information

Supplementary Information.
