# Supplementary material for: NUMB suppression by miR-9-5P enhances CD44+ prostate cancer stem cell growth and metastasis
Source: Sci Rep. 2021 May 27;11:11210. doi: 10.1038/s41598-021-90700-x (PMC8160147; doi:10.1038/s41598-021-90700-x)
Supplement: Supplementary file 1 — Supplementary Information. [file 41598_2021_90700_MOESM1_ESM.docx]

**Online Supplement**

**NUMB suppression by miR-9-5P enhances CD44^+^ prostate cancer stem cell growth and metastasis**

Xuan Wang^2^, Jun Cai^3^, Lei Zhao^1^, Dejun Zhang^1^, Guojie Xu^1^, Jianli Hu^1^, Tao Zhang^1^, Min Jin^1^

^1^ *Cancer Center, Union Hospital, Tongji Medical College, Huazhong University of Science and Technology, Wuhan 430022, China*

^2^*Wuhan Pulmonary Hospital, Wuhan Institute for Tuberculosis Control, Wuhan 430030, Hubei, China*

^3^*Department of Oncology, First Affiliated Hospital of Yangtze University, Hubei, Jingzhou 434000, China*

**Supplemental Figures**


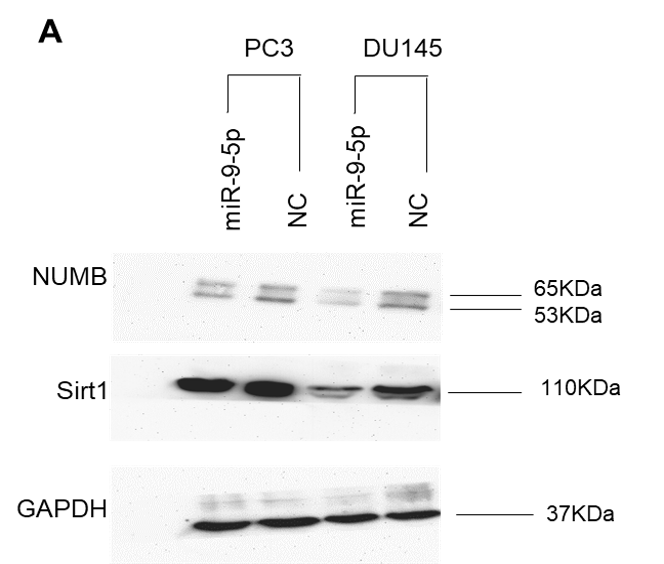


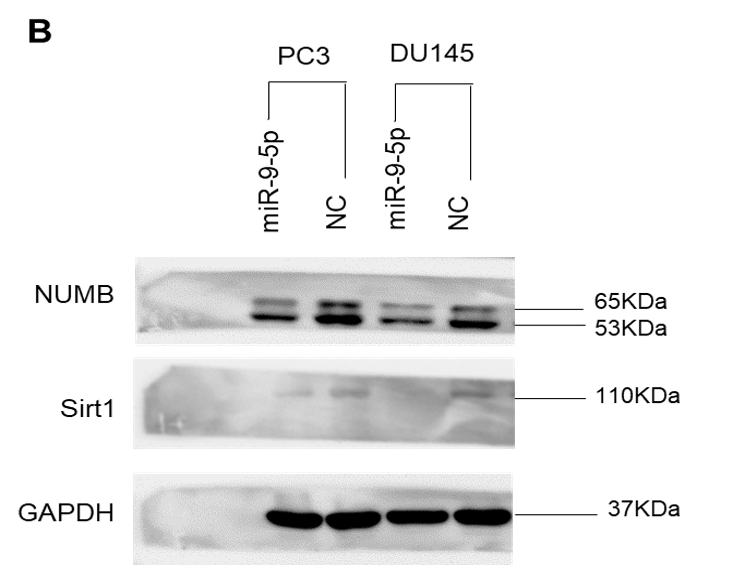


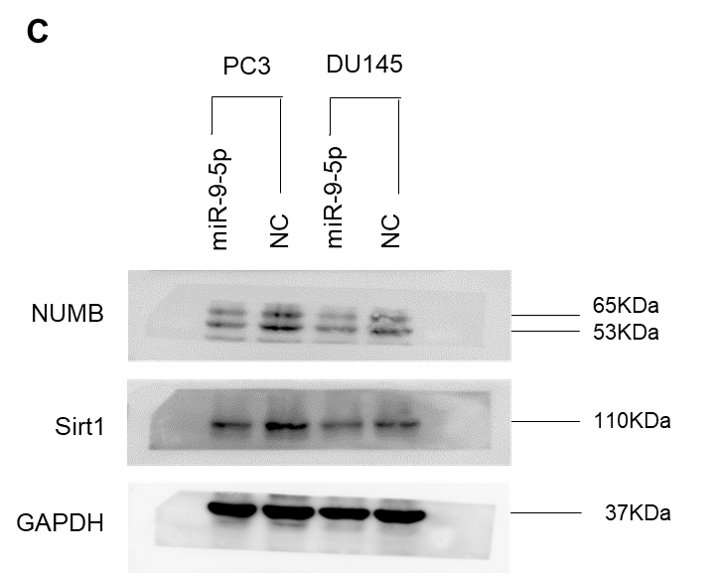


**Figure S1. Primary western blot images of NUMB, Sirt1 and GAPDH.** A representative of 3 repeat experiments of Western Blot by using independent sample sets is presented in PC3 and DU145 cells overexpressing miR-9-5p（A-C）.


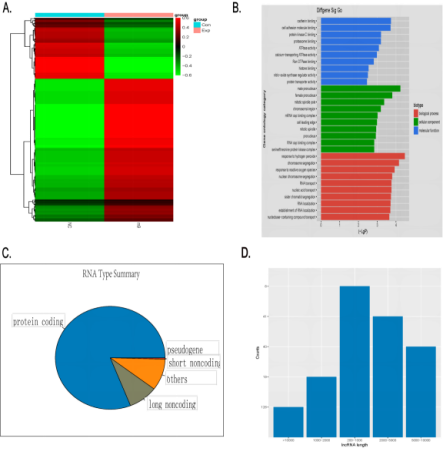


**Figure S2. The expression analysis was undertaken by mRNA-seq in PC3 cells.** (A) Differential gene expression was visualized in a heatmap from RNA-seq analysis of PC3 cells between NC and miR-9-5p mimic transfection. Red represents a high expression value of the differentially expressed genes, and green represents a low expression value of the differentially expressed genes in the grouping samples. (B) Functional enrichment analysis of all differentially expressed genes based on GO analysis. C. Summary of the classification statistics of the differential types of transcripts. D. The length of the target lncRNAs regulated by miR-9-5p.


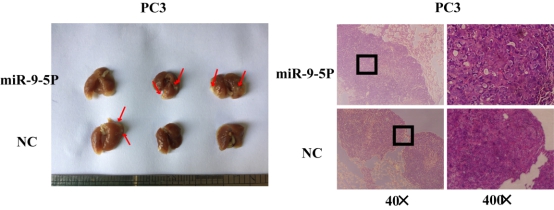


**Figure S3. MiR-9-5p was associated with tumor pulmonary metastasis in the tail vein injection model.** (A) Images shown are the lung tissue harvested 48 days after injecting the PC3 cells transfected with miR-9-5p mimic or mimic NC, and arrows indicate visible lung metastasis nodules. (B) HE staining of lung metastatic nodules from the mice 42 days after injection of the miR-543 mimic- and NC-transfected PC3 cells.
